# The Edinburgh Type 2 Diabetes Study: study protocol

**DOI:** 10.1186/1472-6823-8-18

**Published:** 2008-12-11

**Authors:** Jackie F Price, Rebecca M Reynolds, Rory J Mitchell, Rachel M Williamson, F Gerald R Fowkes, Ian J Deary, Amanda J Lee, Brian M Frier, Peter C Hayes, Mark WJ Strachan

**Affiliations:** 1 Division of Community Health Sciences and Centre for Population Health Sciences, University of Edinburgh, Edinburgh, UK; 2 Centre for Cardiovascular Sciences, Queen's Medical Research Institute, University of Edinburgh, Edinburgh, UK; 3 Metabolic Unit, Western General Hospital, Edinburgh, UK; 4 Psychology in the School of Philosophy, Psychology and Language, University of Edinburgh, Edinburgh, UK; 5 Department of General Practice and Primary Care, University of Aberdeen, Aberdeen, UK; 6 Department of Diabetes, Royal Infirmary of Edinburgh, Edinburgh, UK; 7 Centre for Liver and Digestive Disorders, University of Edinburgh, Edinburgh UK

## Abstract

**Background:**

Risk factors underlying the development and progression of some of the less well-recognised complications of type 2 diabetes, including cognitive impairment and non-alcoholic fatty liver disease, are poorly understood. The Edinburgh Type 2 Diabetes Study was established in 2006 in order to investigate the role of potential risk factors in these complications, as well as to further investigate mechanisms underlying the development and progression of micro and macrovascular disease in type 2 diabetes.

**Methods and design:**

The study is designed as a prospective cohort study. Participants recruited at baseline (2006–2007) constitute 1066 men and women aged 60 to 75 years with established type 2 diabetes, living in the Lothian region of central Scotland. Subjects underwent detailed cognitive and physical examination, the latter including measures of micro- and macro-vascular disease, glycaemic control, body fat composition and plasma inflammatory markers, cortisol, lipids and liver function tests. Participants were re-examined after one year with hepatic ultrasonography and additional measures of vascular disease. This paper reports the methods of recruitment to the study and examinations performed at baseline and one year. Follow-up cognitive, vascular and liver assessments are scheduled for 2010–2011 when subjects will have been in the study for 4 years.

**Discussion:**

This study will provide a wealth of epidemiological and biomarker data that should be invaluable in the identification of potentially modifiable, causal risk factors for diabetes-related cognitive impairment, liver dysfunction and vascular disease, which can be targeted for the development of preventive and therapeutic interventions.

## Background

Type 2 diabetes currently affects around two million people in the UK and approximately 10% of people aged over 65 years. The prevalence of the condition is predicted to double over the next 20 years, with a particular increase in elderly people [1]. Much has been done over the last decade to try to prevent and treat the well-recognised micro- and macrovascular complications of diabetes, including atherosclerotic cardiovascular disease, diabetic retinopathy, nephropathy and peripheral neuropathy. However, morbidity and mortality from vascular disease remains high in older people with type 2 diabetes. In addition, other complications of diabetes are becoming increasingly evident in the ageing diabetic population. These include the deleterious effects of type 2 diabetes on the brain, resulting in a greater prevalence of age-related cognitive impairment and an enhanced risk of age-related cognitive decline, in addition to higher incidences of stroke and dementia [2-4]. Non-alcoholic fatty liver disease, a disorder that encompasses a wide spectrum of hepatic abnormality from fatty infiltration and steatosis to steatohepatitis, fibrosis and ultimately cirrhosis, is increasingly recognised to be associated with the diabetic condition, potentially affecting up to 90% of people with established type 2 diabetes [5-7]. Relatively little is known about the risk factors associated with the development and progression of these less prominent diabetes-related complications, although factors such as poor glycaemic control, increased inflammation, abnormal glucocorticoid metabolism and microangiopathy may be important [8-12]. Detailed information on potential risk factors is crucial to identify causal and modifiable risk factors that can be targeted for the development of appropriate preventive and therapeutic interventions, in addition to helping to identify patients who are at increased risk of developing complications.

The aims of this project were:

1. To determine the association between potentially modifiable risk factors (including microvascular disease, inflammatory mediators and hormones of the hypothalamic-pituitary-adrenal (HPA) axis) and cognitive decrements in people with type 2 diabetes.

2. To determine, in older people with type 2 diabetes, (i) the prevalence of Non-Alcoholic Fatty Liver Disease (NAFLD), (ii) clinical factors that might permit early detection of people at increased risk of developing NAFLD and (iii) potentially causal risk factors for the development and progression of NAFLD.

3. To identify circulating biomarkers and other risk factors which (i) predict the development of symptomatic and asymptomatic micro- and macrovascular disease, (ii) are associated with progression of these complications and/or (iii) have a potentially causal role in their development.

4. To establish a well-characterised and compliant population sample with extensive phenotyping and the potential for genotyping, which can be used as the sampling frame for subsequent nested case control studies (including neuroimaging), and as a replication population for findings arising from genome-wide association studies.

## Methods and design

The study was designed as a population-based, prospective cohort study. Ethical permission was obtained from the Lothian Medical Research Ethics Committee.

### Study Population

#### Sampling frame

With the permission of the Lothian Diabetes Services Advisory Group and the Caldicott Guardian for NHS Lothian, patients recorded as having type 2 diabetes were selected from the Lothian Diabetes Register (LDR). The LDR is a computerised database, which was established in 2001, and contains clinical details on over 20,000 patients with known type 2 diabetes living in Lothian, Scotland. Comparison of age-sex specific prevalences of diabetes recorded on the LDR with those from other data sources in Scotland suggests that the LDR captures almost everyone with diagnosed diabetes in Lothian (Dr Sarah Wild, personal communication).

#### Confirmation of diagnosis of type 2 diabetes

Individual patients are recorded on the LDR once the diagnosis of diabetes has been confirmed according to WHO criteria. Further classification of the patient as suffering from type 2 diabetes is made by medical staff in individuals who are not insulin deficient (as indicated by the presence of ketonuria or ketonaemia) and who have no evidence of secondary diabetes (endocrinopathies or exocrine pancreatic insufficiency) or a genetic aetiology (e.g. Maturity Onset Diabetes of the Young). In order to confirm the diagnosis of type 2 diabetes and exclude individuals erroneously recorded as such on the LDR, the study team reviewed data on potential participants. For the purposes of the present study, the diagnosis of diabetes was accepted in any individual treated with oral anti-diabetic agents and/or insulin, and in any subject treated with dietary modification alone whose HbA1c was > 6.5% at the research clinic. The clinical records of all subjects treated with dietary modification alone and with an HbA1c ≤ 6.5% at the research clinic were reviewed by a consultant diabetologist (MS) to ensure that the diagnosis of diabetes was robust. The clinical records of individuals who either:(i) started on insulin within one year of diagnosis of diabetes, (ii) reported evidence of pancreatic surgery/disease at the research clinic or (iii) were treated with insulin and were aged < 35 years at diagnosis were also reviewed. Such individuals were considered to be at the greatest risk of mis-classification. Any subject in whom it was not possible to confirm a clinical diagnosis of type 2 diabetes by review of hospital and/or GP records was excluded.

#### Exclusion criteria

Other exclusion criteria were, (i) non-English speakers (since fluent English is required for some of the cognitive tasks), (ii) corrected visual acuity worse than 6/36 for distance vision or unable to read large print text (as at least moderate visual function is required to complete some of the cognitive tasks), (iii) unwilling to give consent (or judged by clinical research staff to be unable to give fully-informed consent) (iv) physically unable to complete the clinical and cognitive examination.

#### Power and sample size

The aim was to recruit 1000 subjects in order to provide around 90% power at the two-sided 5% level of significance to detect a Pearson correlation coefficient of ≥ 0.10, between continuous outcome measures (e.g. cognitive test scores) and predictor variables. Allowing for deaths and drop-outs during follow-up, it was estimated that a sample size of 800 would retain 90% power to detect a correlation coefficient of ≥ 0.12 between risk factors and outcome measures. It was also estimated that with this sample size and with the same levels of power and significance, it would be possible to detect any risk factor that contributed 1% or more to the variance in outcome, both at baseline and at follow-up.

### Subject Recruitment

**Figure 1:**
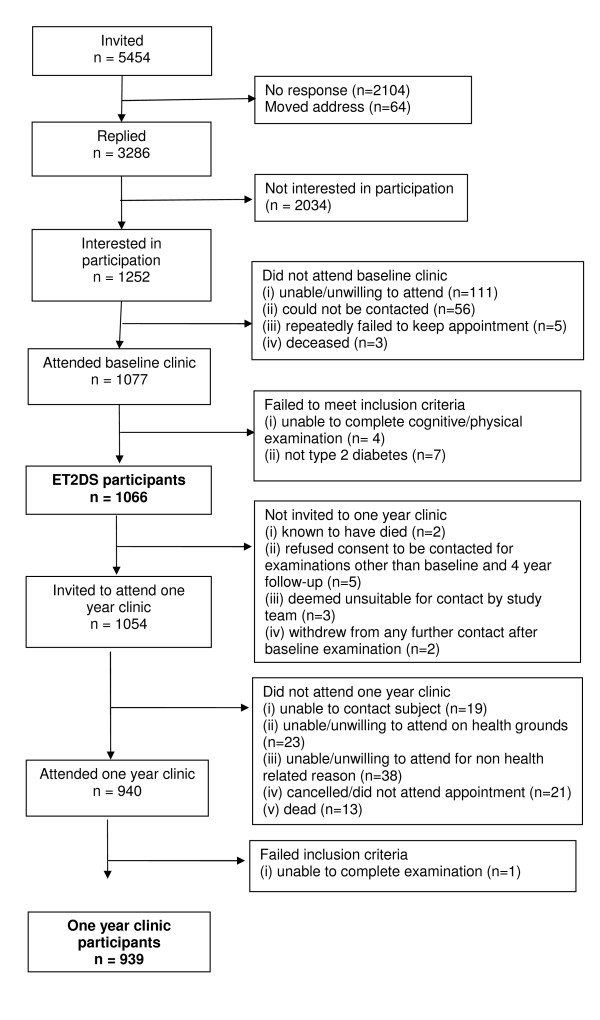
Recruitment and participation in the Edinburgh Type 2 Diabetes Study (baseline and year 1 examinations).

People aged between 60 and 74 years on 1^st ^August 2006 were selected by sex and 5-year age bands from computer-randomised lists of eligible subjects extracted from the LDR. Between 20^th ^June 2006 and 1^st ^June 2007, 5454 invitations to participate in the study were mailed by the custodians of the Lothian Diabetes Register. Of these, no response was received from 2104, and a further 64 were returned as being unknown at the address recorded on the LDR. Of the 3286 individuals who replied, 1252 expressed interest in participating in the study (Figure 1). Another randomly selected subject from the same sex and 5-year age band replaced subjects not replying to the invitation letter or refusing to participate. To preserve patient confidentiality, only the names and addresses of subjects who expressed interest in participating were forwarded to the ET2DS team, precluding follow-up of individual non-responders. Each person expressing an interest in the study was sent an appointment for the research clinic, together with a questionnaire to be completed and returned to the research team. Strenuous efforts were made to ensure that anyone agreeing to participate was actually assessed, including payment of travel expenses, provision of transport to and from the clinic if required, a choice of days to attend the clinic, multiple contact attempts from the ET2DS team by both telephone and mail, and reminder telephone calls on the day preceding clinic appointments. Of the 1252 subjects who expressed interest, 1077 attended the baseline research clinic: the ET2DS team were unable to re-contact 56 individuals, 111 were unable or unwilling to attend for clinical examination when invited, five repeatedly failed to attend their research clinic appointment and three had died. Of the 1077 subjects attending the baseline clinic, four were subsequently excluded from the study because they were unable for physical or emotional reasons to complete the cognitive or physical examinations. In addition, seven subjects were excluded as they did not meet the criteria for type 2 diabetes after detailed review of hospital and primary care notes, searching of electronic databases and, where necessary, discussion with the general practitioner. Of these seven subjects, two diet-treated patients with HbA1c < 6.5% at the research clinic could not be confirmed as having diabetes (from a total of 70 subjects who were reviewed for this reason), four subjects were felt to have type 1 diabetes (from a total of 25 subjects reviewed due to starting on insulin within 1 year of diagnosis or being treated with insulin and diagnosed under the age of 35 years), and one subject had a previous pancreatic neuroendocrine tumour (from a total of 11 subjects reviewed due to reporting prior pancreatic disease). This left 1066 subjects who were both willing and eligible to take part in the ET2DS (Figure 1).

### Schedule of data collection (Figure 2)

**Figure 2:**
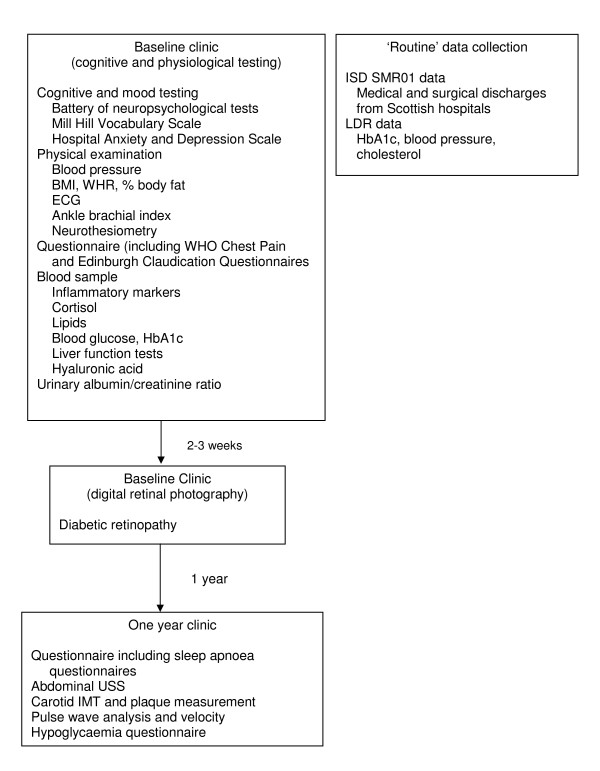
Data collection at baseline and one year research clinics in the Edinburgh Type 2 Diabetes Study.

#### Baseline visit

At baseline and following an overnight fast, all subjects attended a dedicated research clinic where they gave written informed consent for participation in the study. Each subject provided a urine specimen, underwent venepuncture, and, following breakfast, completed a cognitive and physical examination (predominantly for cardiovascular risk factors and vascular disease). The physical examination was undertaken by one of six specially trained research nurses and measurement technicians, using pre-specified standard operating procedures and a specially designed form for data collection. Subjects also returned their completed questionnaires, including questions on demographic characteristics, educational attainment, diabetes history and treatment, cardiovascular disease and other co-morbidities, medications, alcohol intake, smoking habits, employment/occupation, stress, satisfaction with life, subjective social status and personality. Approximately two to three weeks after their initial visit, subjects returned to a separate research clinic for digital retinal photography.

#### 'Routine' data collection

At baseline, data were collected for all participants on discharges from non-psychiatric, non-obstetric wards in Scottish hospitals between 1981 and September 2007 using record linkage to the SMR01 scheme (acute hospital admissions) at the Information and Services Division of NHS Scotland [13]. These data were used to supplement self-reported history of cardiovascular disease, liver conditions and diabetic complications. Selected primary and secondary care data held on the Lothian Diabetes Register (LDR) were also retrieved for participants to provide serial historical data for key biochemical and clinical variables, such as HbA1c, blood pressure and serum cholesterol levels. Non-identifiable data on non-participants were also collected from the LDR, to enable comparison of characteristics with study participants and therefore assess the representativeness of the study population.

#### One year visit

All participants who were alive and who had consented (at baseline) to be contacted about additional studies, were invited to return for a further examination, one year after their recruitment into the study. This visit was primarily for hepatic assessment, including abdominal ultrasound examination, but also enabled further vascular phenotyping (carotid intima media thickness (IMT), carotid plaque assessment, pulse wave velocity and analysis). A total of 940 subjects attended for this examination. Of the remaining 126 original ET2DS participants, 17 had died or had withdrawn from further contact since baseline, 8 had either indicated that they did not wish to be re-contacted for this examination when seen at baseline or were deemed unsuitable for contact by the study team, 61 were unable or unwilling to attend when contacted by letter and/or telephone and 40 either could not be contacted or did not attend their clinic appointment (Figure 1). In addition to the abdominal ultrasound, vascular examinations and venous blood sampling, all of which were performed after a fast of at least four hours, a second questionnaire was completed, including updated questions on alcohol intake, medications and history of liver disorder, Berlin and Epworth sleep apnoea questionnaires [14,15], and questions relating to diabetes healthcare provision in Lothian. Subjects were also provided with short self-assessment questionnaires on which to record the occurrence of severe hypoglycaemic episodes over the subsequent six months.

### Cognitive testing

At baseline, a battery of psychometric tests, which provides a comprehensive and validated assessment of cognitive functions and mood states, was administered. Individual testers were trained and then observed for validation by one investigator (ID). Executive function was assessed with the Borkowski Verbal Fluency Test (VFT), which requires participants to generate as many words as possible with a specified initial letter (C, F and L), within a one minute period for each letter (proper nouns disallowed and repeated words scored only once) [16]. Immediate and delayed verbal declarative memory was assessed using a participant's score on the Logical Memory subtest of the Wechsler Memory Scale 3^rd ^Edition (WMS-III^UK^) [17]. This involves the immediate recall of a story with 25 elements ("story units"), which is read aloud. Participants are informed that they will be questioned about the story again later, and delayed recall is recorded in the same way after approximately 40 minutes. The score is based on the number of story units recalled correctly. Non-verbal memory was assessed using the Faces subtest of the WMS-III^UK^. Subjects are shown a series of pictures of faces and then, in subsequent immediate and delayed tests they are asked to select these faces from a larger series of pictures. As a measure of mental flexibility, the Trail Making Test [18] was administered and the time taken to complete part B was used in the subsequent analysis. Subjects completed several subtests of the Wechsler Adult Intelligence Scale 3^rd ^Edition (WAIS-III^UK^) [19]. The Digit Symbol Coding subtest was used as a measure of speed of information processing – the number of symbols matched correctly to their corresponding numbers in 120 seconds was recorded. The Letter-Number Sequencing subtest was used to assess working memory – participants listened while the tester read mixed, and increasingly longer, strings of numbers and letters. They repeated them to the tester, with the numbers first, in numerical order, and then the letters in alphabetical order. In each item of the Matrix Reasoning subtest (non-verbal reasoning), participants examined a pattern arrayed in matrix with a piece missing. The elements of the matrix are arrayed according to rules. The task is to work out the rules, apply them to find out what the missing piece should look like, and choose the correct piece from the answer options.

All subjects were asked to complete a combined version of the Junior and Senior Form A synonyms of the Mill Hill vocabulary scale [20], an indicator of prior ('best ever' or pre-morbid) cognitive ability which changes very little with age [21,22]. Subjects were presented with a word and asked to identify the closest synonym from six given alternatives. The Mini Mental State Examination [23], often used as a 'screen' for dementia, was included as a general mental assessment.

The Hospital Anxiety and Depression Scale (HAD A and HAD D respectively) [24] was also used for the assessment of mood states, which can affect performance on the cognitive tests. Corrected visual acuity, near vision and capillary blood glucose were also measured before proceeding to cognitive testing. A blood glucose > 4 mmol/l was required, or subjects were asked to return on another day for cognitive testing.

### Assessment of macrovascular disease and cardiovascular risk factors

#### Questionnaire

In the questionnaire applied at baseline, participants answered questions on medical diagnoses and/or treatment (medical or surgical) for angina, coronary heart disease/myocardial infarction, stroke, peripheral arterial disease, carotid stenosis, hypertension and hypercholesterolaemia. Details on the year of diagnosis or event, and hospital or general practice attended, were collected to enable further validation of diagnoses after comparison with ISD and LDR data. Subjects also completed a standard smoking history questionnaire and the WHO Chest Pain [25] and Edinburgh Claudication Questionnaires [26].

#### Physical examination

Systolic and diastolic brachial blood pressures were measured in the right arm to the nearest 2 mmHg with subjects in the supine position and with the arm resting at the level of the mid-sternum, using a standard stethoscope and an aneroid, 6 inch dial, desk standing sphygmomanometer (Acceson™, AC Cossor & Son (Surgical) Ltd, Harlow, UK). To assess body mass index, standing height was measured to the nearest mm, without shoes, using a wall-mounted vertical rule. Weight was assessed to the nearest 0.1 kg without outdoor clothing or shoes using SECA 761 electronic weighing scales. For waist:hip ratio (WHR), waist circumference was measured during exhalation at the level midway between the lower rib margin and the iliac crest (pre-marked at the mid-axillary line on each side of the subject), with the subject standing with their feet 30 cm apart and with their hands by their sides. Hip circumference was taken with the subject in the same standing position, by wrapping the tape measure around the buttocks and lowering or raising the tape until the maximum circumference was located and the tape fitted comfortably. Both measurements were made using a non-expandable tape measure, and the average of two readings taken to the nearest 0.5 cm was taken as the final measurement. Body fat percentage was taken as the average of three consecutive readings (to the nearest 0.1%), using an OMRON BF306 Body Fat Monitor (OMRON Healthcare (UK) Ltd., Henfield, UK). The monitor was pre-set to the correct weight, age and gender for each subject. Subjects stood with their feet 30 cm apart, with their arms at 90° to their trunk and with the palms of each hand placed firmly on the top and bottom electrodes.

A resting 12-lead electrocardiogram was recorded according to standard procedures (Marquette MAC 1200 machine) and coded using the Minnesota coding system by clinical members of the research team  (MS, RR, RW). Right and left brachial, posterior tibial and dorsalis pedis systolic pressures were recorded with the subject in the supine position and after at least 5 minutes rest, using the aneroid sphygmomanometer and a doppler probe (Dopplex^® ^advanced pocket Doppler, Huntleigh Healthcare Ltd., Cardiff, UK). The ankle brachial pressure index (ABI) was calculated for each subject by dividing the lowest of the ankle pressures by the higher of the two arm pressures. To assess inter-observer variation in ABI (and WHR), a total of 20 subjects had repeat measurements of waist and hip circumferences and ankle and brachial pressures, done on a single day by all six of the clinic staff, independently.

Bilateral carotid IMT was measured in the supine position with the neck extended and the chin turned contralateral to the side being examined. A Sonoline Elegra Ultrasound Imaging System (Sieman's Medical Systems Inc, Washington, USA), software version 6, was used. A high frequency linear transducer (8–12 MHz), set up for maximum resolution, was orientated so that the back wall of the common carotid artery was parallel to the transducer face and a double line was clearly seen. IMT was measured on the far wall of the artery, 1–2 cm below the carotid bifurcation and in an area free of plaque. The mean of three measurements (to the nearest 0.1 mm) from three separate images was taken. A longitudinal image was also stored for subsequent off-line serial IMT measurements. The common, internal and external carotid arteries were examined for the presence or absence of plaque. Plaque was defined as a focal structure encroaching into the arterial lumen of at least 0.5 mm or 50% of the surrounding IMT value, or with a thickness > 1.5 mm as measured from the media-adventitia interface to the intima-lumen interface. Plaque at, or within 2 cm, of the bifurcation was recorded bilaterally, together with the maximum thickness of the plaque and the presence or absence of echoluscent plaques. Echoluscent plaque was recorded where one or more plaques appeared as black or almost as black as flowing blood (compared with echogenic plaque which appeared white or almost white, similar to the far wall media-adventitia interface). The presence of heterogeneous plaque was recorded if there were one or more plaques in which echogenicity of more than 20% of the plaque area differed substantially from the echogenicity of the rest of the plaque. Video images were stored for further assessment, including grading of echogenicity.

Pulse wave analysis and velocity are established measures of arterial stiffness whose measurement is increasingly standardised [27]. Measurements were performed after subjects had been supine for at least 25 minutes. Systolic and diastolic brachial blood pressures were measured twice using either the aneroid, 6 inch dial, desk standing sphygmomanometer or a mercurial sphygmomanometer (W.B.I.C, Wenzhou, China) and the mean of each taken. All measurements were taken from the left arm unless this was undesirable or impossible (e.g. previous stroke or amputation affecting left arm) in which case the right side was used. Pulse wave analysis was determined by applanation tonometry of the radial, carotid and femoral arteries using a high-fidelity micromanometer (SPT-301B; Millar Inc, Texas, USA) and the SphygmoCor™ system 8.0 version (AtCor Medical Pty Ltd, New South Wales, Australia). Data from the radial artery pulse wave were collected directly into a computer and, after 10 seconds of sequential waveforms had been acquired, an averaged peripheral waveform was generated. A corresponding averaged central pressure waveform was then generated, using a validated transfer function. From this, central augmentation index (both unadjusted and adjusted to a standard heart rate of 75 bpm) and central aortic blood pressure were determined using the integral software. For measurement of pulse wave velocity the arterial pulse waveform was recorded as above at the common carotid and femoral arteries. The time delay between the arrival of the foot of the pulse wave at the two points was obtained by gating to the peak of the R-wave on electrocardiogram. Separation of the pulse waveforms was defined as the difference between the distances from the sternal notch to the carotid measurement site and from sternal notch to the femoral measurement site. Pulse wave velocity was calculated as distance/time (m/s). All measurements were made in duplicate and the mean values calculated. If there was variation in the augmentation index of greater than 5% or in pulse wave velocity of greater than 0.5 m/s, a third reading was obtained if possible. If the latter was within 5% or 1.0 m/s of one of the first readings then the mean of these was used; otherwise the mean of all three readings was used. If only one reading was obtained then this alone was used.

### Assessment of microvascular disease

Assessment of diabetic retinopathy by digital retinal photography was used as the most sensitive and specific measure of generalised microvascular disease. Standard 7-field digital retinal colour photographs of both eyes were taken at 45% by a single specially trained medical photographer, following pupillary dilatation and using a high resolution digital retinal camera (TOPCON TRC-50FX). Retinopathy was graded using the Early Treatment of Diabetic Retinopathy Scale modification of the Airlie House Classification scheme, which assesses the level of retinopathy for each eye [28,29]. This grading system has been used extensively in clinical and epidemiological studies of diabetic retinopathy, including the Diabetes Control and Complications Trial and the UK Prospective Diabetes Study, to assess baseline status of retinopathy and progression of disease. Two dedicated optometrists graded images independently and discrepancies in coding between the graders were resolved in the first instance by discussion between the graders. Unresolved discrepancies at this point were reviewed and arbitrated upon by a consultant ophthalmologist.

Assessments of peripheral neuropathy and nephropathy were undertaken as secondary measures of microvascular disease. Hand-held neurothesiometer readings were taken from the apex of the big toe (Horwell Neurothesiometer, Scientific Laboratory Supplies Ltd, Nottingham, UK), with subjects sitting or lying, feet elevated and eyes closed. Following a test procedure on the subject's hand, the minimum vibration threshold at which the subject was aware of vibration sensation was recorded to the nearest 0.5 volts. The means of three recordings from each foot were taken as the final measurements. Albuminuria was measured using the urinary albumin/creatinine ratio in an early-morning specimen of urine.

### Liver assessment

At baseline, liver function was assessed by measurement of plasma liver function tests (LFTs) (details below) and hyaluronic acid. In addition a full history of previous liver disease, alcohol intake and use of prescription medications was obtained by questionnaire. At one year, LFTs were repeated, history was updated by questionnaire and a more detailed examination was undertaken using ultrasonography. All ultrasound examinations were performed by a single ultrasonographer who was unaware of the clinical and laboratory results of the participants, on a Sonoline Elegra Ultrasound Imaging System (Sieman's Medical Systems Inc, Washington, USA), software version 6, using a 3.5 MHz transducer. A phantom (411 LE 0.5, GAMMEX rmi Ltd, Nottingham, UK) was scanned multiple times using the different pre-sets prior to the study to ensure continuity of image contrast at follow-up and the appropriate pre-set was chosen for each subject and noted on the ultrasound data collection sheet. Three graders (one sonographer, one specialist registrar in radiology and one consultant radiologist) independently graded the liver for evidence of fatty infiltration using accepted criteria including a bright hepatic echo pattern (compared with the right kidney), increased attenuation of the echo beam and loss of intrahepatic architectural details. Participants received an overall grading of "normal", "indeterminate", "mildly fatty" or "severely fatty". Evidence of more advanced forms of liver disease, including cirrhosis and features of portal hypertension was also sought systematically. In addition the gallbladder, common bile duct, spleen, pancreas, kidneys and aorta were examined. A subgroup of 60 subjects with the full range of liver ultrasound findings underwent detailed magnetic resonance imaging and spectroscopy to quantify liver fat and help validate the ultrasound findings.

Individuals with an abnormal liver ultrasound scan and/or abnormal liver function tests underwent further serological assessment to investigate causes of abnormal liver function, including hepatitis B and C serology, ferritin, anti-smooth muscle and anti-mitochondrial antibodies, anti-nuclear factor and alpha fetoprotein. Individuals with abnormal tests and/or significant derangement of liver function were referred to a specialist hepatology clinic for further investigation and management.

### Blood samples

At baseline, venous blood sample were taken after an overnight fast for DNA extraction, white blood cell immortalisation, measurement of plasma c-reactive protein, fibrinogen, interleukin-6, tissue necrosis factor-α, cortisol and cortisol binding globulin, creatinine, eGFR, total cholesterol, high density lipoprotein cholesterol, blood glucose, HbA1c, total protein, aspartate amino transferase, alanine amino transferase, alkaline phosphatase, gamma glutamyl transferase, bilirubin, albumin, full blood count and hyaluronic acid. At one year, venous blood samples were taken (after a four-hour fast) for uric acid, total cholesterol, low and high density lipoprotein cholesterol, triglycerides, plasma glucose and repeat LFTs and HbA1c. At both baseline and 1 year, plasma was stored for future biomarker measurements.

### Data entry, checking and storage

Data from the baseline questionnaire and data collection forms were coded and entered onto a master Microsoft Access database. The results of plasma assays were entered onto the same master database, either from paper records (biochemistry and haematology) or from electronic files provided by the participating laboratories. For retinopathy grading, data on individual retinal characteristics for each relevant photographic field were entered directly onto a specially designed spreadsheet (Microsoft Excel). The overall retinopathy grade for the right and left eyes for each subject was calculated automatically and these results were transferred to the master database. The majority of data from paper records were double entered and discrepancies resolved by reference to the original paper documentation. For the remaining data a random sample of records was double-checked. A small amount of baseline data that was found to be missing was collected at the one year clinic (primarily clarification of incomplete or inconsistent personal and clinical details) and used to update the master database.

Questionnaire data and plasma assays collected at the one year clinic visit were entered initially onto a separate Microsoft Access database and checked for inconsistencies prior to transfer onto the master database. Data from the abdominal ultrasound scan, carotid IMT and assessment of plaque were entered onto a third Microsoft Access database at the time of grading and these were transferred directly onto the master database. Pulse wave measurements were exported to a Microsoft Excel file and from there entered onto the master database.

All data, including the master database, the retinal photographs and carotid and abdominal ultrasound scans were stored securely on dedicated university computers and backed up on a dedicated university server.

## Discussion

The Edinburgh Type 2 Diabetes Study is a large prospective cohort study that has the potential to identify factors that are important for the development and progression of less well-characterised complications of type 2 diabetes. The study protocol is being reported at the point at which baseline and one year data have been collected and checked. Analyses are currently underway on this rich dataset to address the objectives described in this report. Further analyses to fully meet these objectives will be conducted after the follow-up assessments of cognitive function, vascular parameters and liver structure and function have been completed in 2010–11.

## Competing interests

The authors declare that they have no competing interests.

## Authors' contributions

The principal investigator on the ET2DS grant was JP, and MS, RR, GF, ID, AL and BF were co-investigators. The principal investigator on the liver sub-study was MS, and JP, RR, BF and PH were co-investigators. The investigators designed the study, with AL providing statistical input. RM and RW collected, entered and checked the data. JP drafted the article, with sections contributed by RW, MS and RM. All authors contributed to revising the article. All authors read and approved the final article.

## Pre-publication history

The pre-publication history for this paper can be accessed here:


